# Reply to Fillmore et al.: Additional evidence of non–SARS-CoV-2 coronavirus cross-immunity

**DOI:** 10.1073/pnas.2215620119

**Published:** 2022-11-07

**Authors:** Matthew D. Solomon, Lawrence Steinman, Vincent X. Liu

**Affiliations:** ^a^Division of Research, Kaiser Permanente Northern California, Oakland, CA 94611;; ^b^Department of Cardiology, Kaiser Oakland Medical Center, Oakland, CA 94611;; ^c^Department of Pediatrics, Stanford University, Stanford, CA 94305;; ^d^Department of Neurology and Neurological Sciences, Stanford University, Stanford, CA 94305;; ^e^Department of Critical Care Medicine, Kaiser Santa Clara Medical Center, Santa Clara, CA 95051

We are glad that our paper inspired Fillmore et al. (1) to report their findings about potential preexisting immunity to common non–SARS-CoV-2 human coronaviruses (HCoVs). Swadling et al. (2) have done extensive studies showing that the proteins involved in the replication of different coronaviruses are highly conserved. Swadling et al. (2) and others (3–6) reinforce the concept that some degree of existing immunity to common coronaviruses is protective to SARS-CoV-2. Our work on a large sample from Kaiser Permanente Northern California, where we care for >4.5 million persons, helps provide some epidemiologic evidence to help explain the molecular immunology (7). The papers fit together to provide an understanding of how populations of individuals gain robust immunity to this family of viruses. However, the findings of Fillmore et al. (1) do warrant a closer examination.

First, the authors examined only a 1-y period during the start of the COVID-19 pandemic to compare incidence rate ratios for the outcome of COVID-19 infection in those with positive HCoV test results vs. those with negative HCoV test results. In our opinion, COVID-19 infection rates are the least meaningful outcome related to the COVID-19 pandemic compared with measurements of COVID-19–related hospitalization, need for the intensive care unit, or mortality. The risk of COVID-19 infection depends upon many factors, including the extent of exposure (which was not measured), which may vary greatly between groups. In addition, the time at risk for patients in the HCoV-positive vs. -negative groups after an HCoV test result is not clarified. Without clarification, there might exist a bias in the results. For example, if those with positive results—of whom there were only 332 of a total of 58,263, 0.5% of all the patients with both SARS-CoV-2 and HCoV tests—had much less time at risk after their HCoV-positive test, this might skew the results.

In addition, the very low rate of HCoV test positivity among those tested is curious. Could it have been a result of the shelter-in-place phenomenon during the first year of the pandemic? Under this policy, social exposures to typical circulating viral illnesses were minimized. Finally, the analysis that compared more distant HCoV exposure 5 y prior to the study period with the risk of COVID-19 infection during the pandemic’s first year requires more detail.

Overall, we appreciate the interest and findings by the authors and encourage them to elaborate further in more detailed analyses.
